# Effect of Ferric Carboxymaltose on Exercise Capacity in Patients With Chronic Heart Failure and Iron Deficiency

**DOI:** 10.1161/CIRCULATIONAHA.117.027497

**Published:** 2017-10-09

**Authors:** Dirk J. van Veldhuisen, Piotr Ponikowski, Peter van der Meer, Marco Metra, Michael Böhm, Artem Doletsky, Adriaan A. Voors, Iain C. Macdougall, Stefan D. Anker, Bernard Roubert, Lorraine Zakin, Alain Cohen-Solal

**Affiliations:** From Department of Cardiology, University Medical Center Groningen, University of Groningen, The Netherlands (D.J.v.V., P.v.d.M., A.A.V.); Department of Heart Diseases, Medical University, Clinical Military Hospital, Wroclaw, Poland (P.P.); Department of Cardiology, University Hospital, Brescia, Italy (M.M.); Universitätsklinikum des Saarlandes, Homburg/Saar, Germany (M.B.); I.M. Sechenov First Moscow State Medical University, Moscow, Russia (A.D.); King’s College Hospital, London, United Kingdom (I.C.M.); Division of Cardiology and Metabolism, Department of Cardiology (CVK) and Berlin-Brandenburg Center for Regenerative Therapies (S.D.A.); Deutsches Zentrum für Herz-Kreislauf-Forschung, Berlin (S.D.A.); Charité Universitätsmedizin Berlin, Germany (S.D.A.); Department of Cardiology and Pneumology, University Medicine Göttingen, Germany (S.D.A.); Vifor Pharma, Glattbrugg, Switzerland (B.R., L.Z.); and Hopital Lariboisiere, University Paris Diderot, UMR-S942, France (A.C.-S.).

**Keywords:** exercise capacity, ferric carboxymaltose, heart failure, iron deficiency

## Abstract

**Background:**

Iron deficiency is common in patients with heart failure (HF) and is associated with reduced exercise capacity and poor outcomes. Whether correction of iron deficiency with (intravenous) ferric carboxymaltose (FCM) affects peak oxygen consumption [peak VO_2_], an objective measure of exercise intolerance in HF, has not been examined.

**Methods:**

We studied patients with systolic HF (left ventricular ejection fraction ≤45%) and mild to moderate symptoms despite optimal HF medication. Patients were randomized 1:1 to treatment with FCM for 24 weeks or standard of care. The primary end point was the change in peak VO_2_ from baseline to 24 weeks. Secondary end points included the effect on hematinic and cardiac biomarkers, quality of life, and safety. For the primary analysis, patients who died had a value of 0 imputed for 24-week peak VO_2_. Additional sensitivity analyses were performed to determine the impact of imputation of missing peak VO_2_ data.

**Results:**

A total of 172 patients with HF were studied and received FCM (n=86) or standard of care (control group, n=86). At baseline, the groups were well matched; mean age was 64 years, 75% were male, mean left ventricular ejection fraction was 32%, and peak VO_2_ was 13.5 mL/min/kg. FCM significantly increased serum ferritin and transferrin saturation. At 24 weeks, peak VO_2_ had decreased in the control group (least square means −1.19±0.389 mL/min/kg) but was maintained on FCM (−0.16±0.387 mL/min/kg; *P*=0.020 between groups). In a sensitivity analysis, in which missing data were not imputed, peak VO_2_ at 24 weeks decreased by −0.63±0.375 mL/min/kg in the control group and by −0.16±0.373 mL/min/kg in the FCM group; *P*=0.23 between groups). Patients’ global assessment and functional class as assessed by the New York Heart Association improved on FCM versus standard of care.

**Conclusions:**

Treatment with intravenous FCM in patients with HF and iron deficiency improves iron stores. Although a favorable effect on peak VO_2_ was observed on FCM, compared with standard of care in the primary analysis, this effect was highly sensitive to the imputation strategy for peak VO_2_ among patients who died. Whether FCM is associated with an improved outcome in these high-risk patients needs further study.

**Clinical Trial Registration:**

URL: http://www.clinicaltrials.gov. Unique identifier: NCT01394562.

## Abstract 2

Supplemental Digital Content is available in the text.

**Editorial, see p 1384**

**Clinical Perspective**

**What Is New?**

- Iron deficiency is increasingly recognized as a serious comorbidity in patients with heart failure and is associated with increased morbidity and mortality.
- In earlier studies, patient treatment with ferric carboxymaltose maltose (FCM, intravenous iron) was shown to decrease symptoms and improve functional capacity.
- In the present study, iron supplementation with FCM led to repletion of iron stores and improvement in measures of disease severity and quality of life.
- A decline in peak VO_2_ in the control group was driven by deaths, with imputed values of 0 in the control group. This decline was not seen in the FCM group.

**What Are the Clinical Implications?**

- Iron supplementation with (intravenous) FCM significantly increased serum ferritin and transferrin saturation.
- The effects of iron supplementation with (intravenous) FCM include improvements in quality of life.
- Further study is needed to determine the impact of intravenous FCM supplementation on functional capacity and clinical outcome.

Iron deficiency is increasingly recognized as an important comorbidity in patients with heart failure (HF) and is present in up to 35% to 50% of patients with HF.^1–5^ Its prevalence is related to the severity of the disease and is a strong and independent predictor of outcome, in both the presence and absence of anemia. Iron deficiency decreases exercise capacity, in both healthy volunteers and patients with chronic HF.^4,6–8^ Iron plays an important role in oxygen transport, not only through hematopoiesis but also in the metabolism of cardiac and skeletal muscles. All these factors play a role in the reduced exercise capacity in HF.

Given the importance of iron deficiency, a clear rationale exists for investigating the effect of correcting this, and a few small studies suggested cause for optimism.^2,7^ More recently, 2 larger randomized controlled trials have been conducted with (intravenous) iron (ie, ferric carboxymaltose [FCM]) in patients with HF with iron deficiency.^9,10^ These studies showed that FCM improved symptoms, quality of life, and functional capacity, irrespective of the presence of anemia. The drug was well tolerated and safe. At 1-year follow-up, FCM was also associated with a reduction in the risk of first hospitalization for worsening HF.^10^

Exercise intolerance is one of the hallmarks of chronic HF, and it is further reduced in the presence of iron deficiency.^2,4,6–8^ Although intravenous iron (FCM) was shown to increase 6-minute walking test distance in patients with HF,^9,10^ this parameter may be subject to some bias. Therefore, investigation of more robust and objective parameters during cardiopulmonary exercise testing (CPET), such as peak oxygen consumption (peak VO_2_),^11^ would seem useful.

The aim of the present EFFECT-HF study (Effect of Ferric Carboxymaltose on Exercise Capacity in Patients With Iron Deficiency and Chronic Heart Failure) was to examine the effect of treatment with intravenous FCM, compared with standard of care, on exercise capacity in patients with symptomatic chronic HF and iron deficiency.

## Methods

### Patient Population

Patients were eligible for the EFFECT-HF study if they were ≥18 years of age, had clinically stable mild to moderate chronic HF (New York Heart Association [NYHA] functional class II–III), and were on optimal background therapy for HF for ≥4 weeks and with no dose changes in the last 2 weeks. Their left ventricular ejection fraction had to be ≤45% and had to be performed in ≤3 months of screening and >3 months after stable β-blocker therapy or device implantation, in particular cardiac resynchronization therapy. Plasma brain natriuretic peptide (BNP) concentration at baseline had to be >100 pg/mL or N-terminal (NT) proBNP had to be >400 pg/mL. Patients were required to have a decreased exercise capacity, as shown by a reproducible peak VO_2_ of 10 to 20 mL/kg/min. Patients also needed to have documented iron deficiency, as defined by a serum ferritin <100 ng/mL or a serum ferritin of 100 to 300 ng/mL in combination with a transferrin saturation (TSAT) <20%.

Patients were excluded from the study if they had a known sensitivity to FCM, a history of iron overload, or received erythropoiesis-stimulating agents, intravenous iron therapy and/or blood transfusions in the 6 weeks before randomization. There was no lower limit of hemoglobin (Hb), but subjects with an immediate need of blood transfusion or an Hb >15 g/dL were also excluded. Patients who had undergone an exercise training program in the previous 3 months, or those planned in the next 6 months, as well as those with an active bacterial infection, those with serious liver disease (transaminases >3 times the upper limit), a known vitamin B12 or serum folate deficiency, or a known human immunodeficiency virus or hepatitis B or C infection were also excluded. Patients with clinical evidence of current malignancy and on renal dialysis were also not included. Patients with unstable angina pectoris, severe valvular disease, or left ventricular outflow tract obstruction; patients with atrial fibrillation or flutter with a ventricular response rate of >100 beats per minute at rest; and patients with recent (<3 months) acute coronary syndrome, coronary artery bypass surgery, percutaneous coronary interventions, transient ischemic attack, or stroke were also excluded. Finally, women who were pregnant or without adequate contraception and subjects with (recent) participation in another study or with low body weight (<35 kg) were excluded. The study followed the principles outlined in the Declaration of Helsinki. Ethical approval was obtained from the Medical Ethics Committee of each participating center, and all subjects gave their written informed consent before enrollment.

### Study Design

The EFFECT-HF study was a prospective randomized controlled, multicenter, open-label trial with blinded end-point evaluation. It aimed to examine the effect of treatment with intravenous FCM, compared with standard of care, on exercise capacity in patients with symptomatic chronic HF and iron deficiency. After an initial screening period (≤12 weeks), eligible subjects were randomized (1:1) to FCM or usual care for a period of 24 weeks. Study visits took place on day 0 (baseline) and at weeks 6, 12, and 24. Within the screening period, the reproducibility of peak VO_2_ had to be confirmed with applicable tests. Subjects randomized to standard of care could receive oral iron at the discretion of their attending physician but were not permitted intravenous iron.

The primary end point was the change in peak VO_2_ from baseline to 24 weeks. Secondary end points included the effect of FCM on hematinic indices (Hb, ferritin, and TSAT), natriuretic peptides (BNP and NTproBNP), NYHA functional class, and patient global assessment. In addition, safety parameters were collected.

The study was designed and conducted by the steering committee, and an independent data safety monitoring board reviewed data on an ongoing basis. The independent Endpoint Adjudication Committee adjudicated all hospitalizations and deaths. All analyses were performed according to a predefined statistical analysis plan validated by the sponsor, Vifor Pharma Ltd. The manuscript was prepared by the principal investigator (D.J.v.V.) and subsequently reviewed by all authors and Vifor Pharma for scientific accuracy, but the authors had final authority. The authors vouch for the accuracy and completeness of the reported analyses. The EFFECT-HF study is registered at Clinical.Trials.gov, NCT01394562.

### Study Drug Administration

Intravenous iron was given as FCM solution (Ferinject/Injectafer, Vifor Pharma) as an undiluted intravenous bolus injection (administered in ≥1 minute) or an infusion. Infusions of 10 or 20 mL (which is the amount of FCM that is equivalent to 500 or 1000 mg of iron, respectively) were administered diluted in ≈100 mL of sterile 0.9% sodium chloride solution and given in ≥6 minutes for 10 mL (infusion of 20 mL diluted in ≈200 mL and administered in ≥15 minutes). Dosing at day 0 and week 6 was based on screening Hb and weight and not on ferritin and TSAT results. On day 0 (baseline), patients with Hb ≤14 g/dL, both < and >70 kg received 1000 mg FCM (20 mL), whereas patients >14g/dL received 500 mg FCM (10 mL). At week 6, patients <70 kg with Hb <10 g/dL received a second dose of 500 mg FCM, whereas patients with Hb ≥10 g/dL received no second dose. At week 6, for patients ≥70 kg, those with Hb <10 g/dL received another dose of 1000 mg FCM, whereas those with Hb 10 to 14 g/dL received 500 mg FCM, and those with Hb >14 g/dL received no additional dose. At week 12, FCM was only administered (at a dose of 500 mg FCM) if serum ferritin was <100 ng/mL or if ferritin was 100 to 300 ng/mL with TSAT <20% (ie, if patients were still iron deficient). No specific treatment advice was given for patients in the standard of care group, and oral iron was allowed.

### Exercise Testing

#### General Organization for Participating Centers

Before the trial, ≥1 investigator from each participating site attended a 1-day session (at the Exercise Laboratory of A.C.-S. in Paris) to homogenize the CPET methods. A video and book summarizing all technical recommendations were provided to all investigators. Before including the first patient in the study, all centers validated the measurement system. All data were reviewed blindly (ie, unaware of treatment assignment) by the core laboratory in Paris.

#### CPET Methodology

CPET was done at screening (<3 months before baseline), baseline, and weeks 12 and 24. To be eligible for the study, patients were required to have a decreased exercise capacity as shown by a peak VO_2_ of 10 to 20 mL/kg/min. All patients underwent gas exchange with breath-by-breath measurements during exercise according to current standards.^12^ Exercise capacity was estimated by 2 tests (completed within 4 weeks of each other and ≥3 days apart) with ≥1 test completed within 4 weeks before baseline visit. An earlier test could be used as test 1 if performed within 4 weeks before screening. The 2 tests were required to have <10% difference in values (and the last CPET was used as the baseline value). If >10% difference, a third test could be performed within 4 weeks and ≥3 days after the second test. For the baseline tests, the respiratory exchange ratio (RER) had to be ≥1.0. Exercise tests were all conducted on a bicycle. A graded protocol was used; after a rest stage of ≥2 minutes, the work rate was increased by 10 W every minute (or with a ramp protocol with a similar increment on load) after a first 20 W stage. Patients were exercised until exhaustion or when an incident led to termination of the test (including serious arrhythmia, sudden drop in BP, angina, and ischemia). Only tests stopped because of fatigue or dyspnea were retained in the analysis. At peak exercise, patients were asked to continue pedaling at a slow pace with a work rate of 20 W during ≥3 minutes. Heart rate was continuously monitored and blood pressure measured every 2 minutes. A standard 12-lead ECG was recorded continuously during exercise.^12^

Gas analyzers were calibrated before each test. Patients were asked to start exercise only when baseline RER was <1, baseline oxygen consumption (VO_2_) was between 3 and 6 mL/min/kg, and ventilation (VE) was between 8 and 12 L/min. Gas sampling was done through a face mask. All tests had to be symptom-limited, with strong encouragement to achieve an RER >1.10 and a Borg rating of perceived exertion >16/20. Only tests with a peak RER >1.0 were accepted at baseline. Only patients with a peak VO_2_ between 10 and 20 mL/min/kg could be included. The VO_2_, carbon dioxide production, and VE were measured breath by breath.

The primary end point was change in peak VO_2_ in ml/min/kg. Peak VO_2_ was the average of the values over the last 20 seconds of exercise. We also measured the slope between minute VE and carbon dioxide production (also named ventilatory efficiency, secondary end point) because this parameter can be assessed even when exercise is submaximal and has important prognostic value similar to that of peak VO_2_.^13,14^

### Other Secondary End Points

Functional class was assessed by the attending physician throughout the study according to the NYHA functional class. Patient global assessment (self-reported) was assessed using a simple score, which consists of a simple judgment indicating improvement, no change, or worsening since the start of the study, using the Likert scale, as previously described^15^ and also used in previous studies with FCM.^9,10^ In short, the effect of FCM on symptoms is reported as an odds ratio for being in a better rank versus the control group, whereby a value >1.0 indicates an improvement. Analyses of the effect of FCM on NYHA class and patient global assessment were conducted with and without imputation of missing values (observed values). BNP and NTproBNP were measured in a core laboratory using standard methodology. Effect of FCM on hematinics was also examined, and safety data were collected.

### Statistical Analysis

The sample size calculation was based on an earlier small focused study^16^ with (another) intravenous iron, in which the difference in peak VO_2_ between active treatment and controls was 2.2±2.1 mL/min/kg. Hence, the steering committee elected a more conservative estimate of a difference of 1.5 and a standard deviation of 2.8 mL/min/kg for the power calculation. Based on these assumptions, a sample size of 75 patients per group (150 total) was needed for 90% power using an alpha of 0.05 (2-sided). The sample size was rounded up to 80 patients per group (160 total) to allow for some loss of information.

Three analysis sets of subjects were considered for the statistical analysis of the study.

A full analysis set (FAS) defined according to the intention to treat principle included all subjects who had ≥1 study drug administration in FCM group, were randomized in usual care (control) group, and had ≥1 efficacy assessment after baseline. The primary analysis of the primary end point, the change from baseline of the weight-adjusted peak VO_2_ at week 24, was performed on the FAS. A peak VO_2_ of 0 was imputed to subjects who died before week 24, and a last observation carried forward (LOCF) imputation was used for subjects without assessment at week 24. An analysis of covariance model, including as covariates baseline-adjusted peak VO_2_, country, and screening hemoglobin (<12 g/dL or ≥12 g/dL), was used to compare treatment groups.

A supportive per protocol (PP) set analysis was applied to assess the robustness or sensitivity of the primary analysis, in which all subjects from the FAS were included who did not have a major protocol deviation, as defined in a Blind Data Review Meeting before the database lock. Also in this set of subjects, LOCF imputation has been used for missing values and 0 values for subjects who died, not including the secondary sensitivity analysis. In addition, assessments of subjects who had a forbidden treatment (blood transfusion, erythropoiesis-stimulating agents) were censored from the day the forbidden treatment was started. Furthermore, subjects were censored for the PP analysis if they had a cardiopulmonary exercise test that was not considered valid (in a blind manner) by the core laboratory (RER <1, wrong exercise protocol).

A safety set included all subjects who had a study drug administration in the FCM group or were randomized in standard of care group (ie, also including patients who had no efficacy assessment after baseline). All-cause mortality and hospitalization for worsening HF and other cardiovascular reasons were analyzed for the safety set. Safety analyses were performed on all subjects who received ≥1 dose of study drug or were randomized to the usual care group.

A number of additional supportive analyses of the primary outcome were also performed on the FAS and the PP set of subjects by (1) imputing worst value across all patients and all time points for missing values because of hospitalization and 0 for patients who died (worst value imputation), (2) using only observed values without any imputation using a repeated measures ANCOVA model for the FAS and PP set, and (3) terms of interaction between pooled country and treatment group as well as interaction between Hb level at screening and treatment group was evaluated.

NYHA classification scores were analyzed over time using a repeated measures polytomous regression and at week 24 with LOCF imputation using a polytomous regression, with multinomial logistic regressions. This analysis was performed on the change of NYHA class compared with baseline and given relative to the standard of care arm as described before.^9,10^

## Results

### Study Population

**Figure 1.**
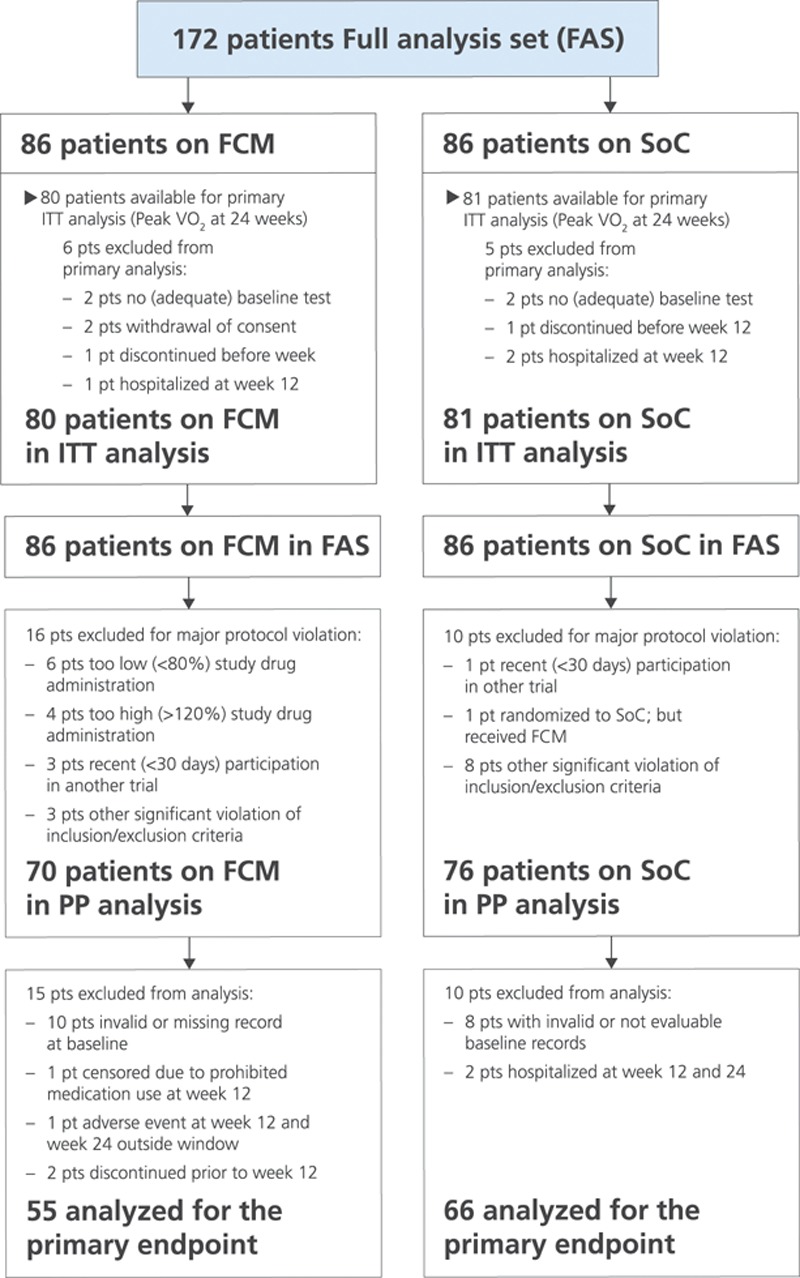
**Consort diagram of the EFFECT-HF study (Effect of Ferric Carboxymaltose on Exercise Capacity in Patients With Iron Deficiency and Chronic Heart Failure).** Enrollment, outcomes, and availability of peak VO_2_ test during the study are depicted. FCM indicates ferric carboxymaltose; ITT, intention to treat; peak VO_2_, peak oxygen consumption; PP, per protocol; and SoC, standard of care.

We enrolled 174 patients from 28 sites across 9 countries (Australia, Belgium, France, Germany, Italy, The Netherlands, Poland, Russia, and Spain). Two patients in the FCM arm did not have an efficacy assessment after baseline, and these 2 were excluded from the FAS. Therefore, the study population consisted of 172 patients (Figure 1); their baseline characteristics are depicted in Table 1. At baseline, the 2 groups were generally well matched. Of the 172 patients, 161 patients could be examined for the primary end point: effect on peak VO_2_ during CPET after 24 weeks (Figure 1). A total of 146 patients were analyzed in the PP population. Of the 86 patients in the usual care group, 29 received oral iron supplementation by their attending physician (because this was allowed according to the protocol).

**Table 1.**
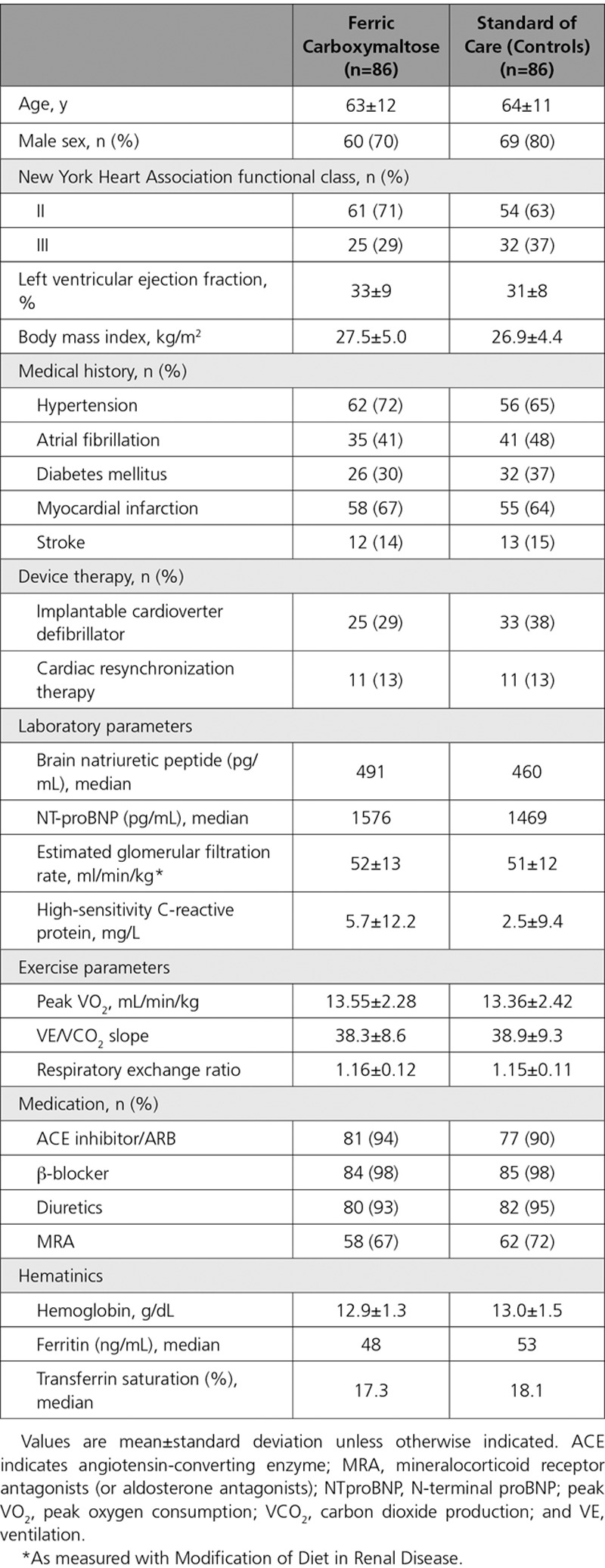
Baseline Characteristics of the Study Patients According to Treatment Group

### Iron Administration and Dosing

At baseline, the 2 groups were similar in terms of Hb, serum ferritin, and TSAT (Table 1). All patients by definition had iron deficiency, but a decreased serum ferritin (<100 ng/mL) was present in 84% of patients and a decreased TSAT (<20%) in 58%. Of the 86 patients in the FCM group, 37 (42%) received only 1 administration, 48 patients (55%) needed 2, and 3 (3%), required 3 administrations. Mean administered dose of iron (FCM) was 1204±391 mg (median dose 1000 mg). After 24 weeks (end of study), all hematinic indices had increased significantly in the FCM group: Hb was 13.9±1.3 g/dL, ferritin was 283±150 ng/mL, and TSAT was 27±8% (all *P*<0.05 compared to baseline).

Patients in the standard of care group also showed small changes in hematinics after 24 weeks (Hb 13.2±1.4 g/dL and ferritin 79 ng/mL; both *P*=NS [not significant] versus baseline), whereas TSAT slightly increased to 20.2% (*P*=0.035 compared to baseline). All changes in the standard of care group were significantly different (*P*<0.05) versus FCM.

### Effect on Exercise Parameters

At baseline, the 2 groups were well matched for peak VO_2_ and VE/carbon dioxide production slope (Table 1).

**Figure 2.**
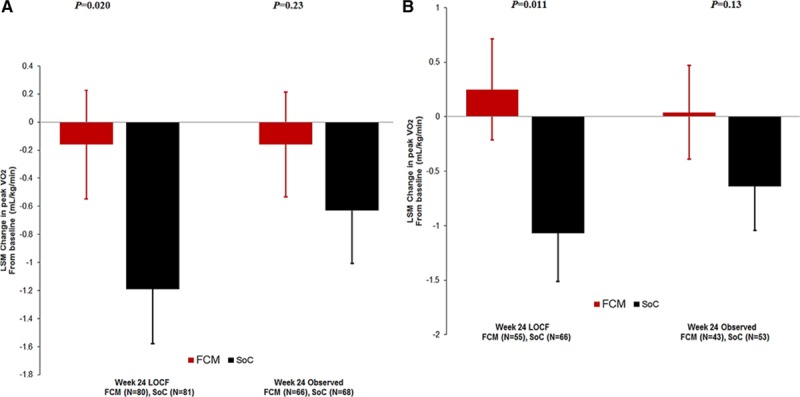
**Effect of peak VO_2_ after 24 weeks.** **A**, Effect of peak VO_2_ after 24 weeks in the whole (FAS) population. **Left**, LOCF (primary end point) is shown. **Right**, observed values (sensitivity analysis) are shown. **B**, Effect of peak VO_2_ after 24 weeks in the PP population. **Left**, LOCF (secondary end point) is shown. **Right**, observed values (sensitivity analysis) are shown. FAS indicates full analysis set; FCM, ferric carboxymaltose; LOCF, last observation carried forward; LSM, least square means; peak VO_2_, peak oxygen consumption; PP, per protocol; and SoC, standard of care.

After 24 weeks, peak VO_2_ (LOCF, primary end point; n=161) had decreased by –1.19±0.389 mL/min/kg in the standard of care group, whereas it was virtually unchanged in the FCM group (−0.16±0.387 mL/kg/min) (the difference of least square means±standard error: 1.04±0.44 mL/kg/min; *P*=0.020 between groups) (Figure 2A, left). Without imputation of deaths, peak VO_2_ at 24 weeks decreased by −0.63±0.375 mL/min/kg in the control group (the difference of least square means 0.48±0.398 mL/kg/min, *P*=0.23 between groups) (Figure 2A, right).

In the PP set (n=146), a similar difference between groups after 24 weeks was observed (ie, +0.25 mL/kg/min for FCM versus −1.10 mL/kg/min in the standard of care group) (difference 1.32±0.51 mL/kg/min, *P*=0.011 between groups; Figure 2B, left), but this was also not statistically significant anymore when no values were imputed (week 24 observed) for the patients who died (*P*=0.13; Figure 2B, right).

At 12 weeks, the change in peak VO_2_ was +0.25 mL/min/kg for FCM versus −0.34 mL/min/kg for the usual care patients (difference 0.45±0.38; *P*=0.24 between groups; observed values).

The VE/carbon dioxide production slope was similar at baseline in the 2 groups and markedly elevated (ie, abnormal). Small changes occurred in both groups after 24 weeks (−1.2 for FCM versus −1.1 for usual care patients), but no difference was found between groups (*P*=0.93). Patients with anemia (Hb <12 g/dL) did not benefit more from FCM than those without anemia (*P* value for interaction 0.758), and in the group of patients treated with FCM, no association was discovered between the change in Hb and the change in peak VO_2_ (for FCM: *r*=−0.0758, *P*=0.51) (Figure I in the online-only Data Supplement).

There was also no influence of where (which country) the patient was enrolled, and there was no difference between men and women in the effect of FCM.

Results of the different supportive sensitivity analyses showed findings that were generally similar using different modeling options, although a less robust effect was noted with no statistically significant difference between groups in the supportive analyses, which used only observed values (ie, those with no imputation for death) (data not shown).

### Follow-Up

During the 24-week study, 4 patients died after 8, 51, 106, and 146 days, all in the usual care group (1 additional patient [usual care] died on day 190, ie, within 30 days of completion of the study.) Two patients died suddenly, 1 was because of worsening HF, and 2 had complications during an episode of worsening HF (1 sepsis, 1 respiratory failure).

A total of 58 hospitalizations occurred during the study in 40 patients (Table 2); 26 of them for worsening HF (13 in each group) in 17 patients (11 patients on FCM and 6 on usual care). Sixteen other cardiovascular hospitalizations occurred in 15 patients. Thirteen of these hospitalizations were observed in 12 patients on FCM, and 3 other cardiovascular hospitalizations occurred in 3 patients in the standard of care group (13 versus 3 hospitalizations, *P*<0.05 between groups). Of these 13 (other cardiovascular) hospitalizations in the FCM group, 3 were related to an implantable cardioverter defibrillator (1 malfunction, 1 elective new implantation, and 1 replacement), 2 patients had an elective coronary angiography (planned before enrollment into the study), and 1 patient underwent an already planned ablation for atrial flutter. In the standard of care group, 1 patient had an appropriate shock for ventricular fibrillation and survived. None of these hospitalizations was considered related to study drug administration by the attending physician.

**Table 2.**
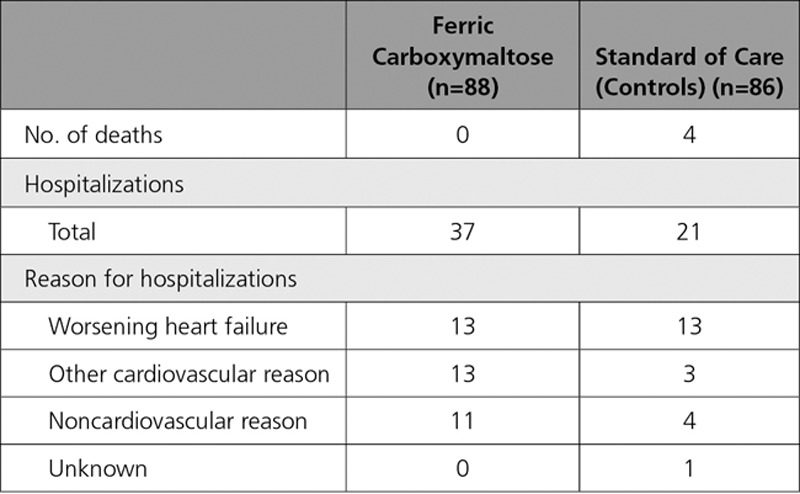
Number of Deaths and Hospitalizations During the Study

In an additional (post hoc) analysis, we compared the number of patients with worsening HF or death. Eleven patients in the FCM were hospitalized for HF, and no deaths occurred. In the standard of care group, 4 patients died during the study (1 of them during a hospitalization for worsening HF), and 5 additional patients were hospitalized for worsening HF; 1 more patient had a cardiac arrest at home with documented ventricular fibrillation, for which he later received an implantable cardioverter defibrillator, which leads to 9+1 patients in the standard of care group.

### Effects on Other Clinical Parameters and Safety

NYHA functional class was not significantly different at baseline in the 2 groups. After 6, 12, and 24 weeks, patients on FCM had improved their NYHA functional class significantly compared with patients in the control group (with imputation; all differences *P*<0.05) (Figure II in the online-only Data Supplement). The effect of FCM on NYHA class without imputation (observed values) was similar and also significantly different from the control group.

Patient global assessment was also similar at baseline and favorably affected by FCM compared with placebo. This difference was not present at 6 weeks but became evident at 12 and 24 weeks (with imputation; *P*<0.05 between groups) (Figure III in the online-only Data Supplement). The results without imputation (observed values) were remarkably similar and also significantly different at 12 and 24 weeks. BNP and NTproBNP were markedly elevated at baseline and were well balanced between groups. No significant effect of FCM compared with usual care was observed for BNP during the study. For NTproBNP, at 6 and 12 weeks no effect versus controls was observed, but at 24 weeks a trend toward a lower increment in NTproBNP in the FCM group was observed that was not statistically significant (+193 pg/mL versus +834 pg/mL for those on usual care; *P*=0.13 with LOCF, FAS population, n=172). In the PP analysis (n=146 patients), this difference reached statistical significance in favor of FCM at 24 weeks (*P*=0.048 between groups).

FCM was generally well tolerated. No hypersensitivity reactions to the drug occurred, and no cases of hypophosphatemia were reported.

## Discussion

Iron deficiency is increasingly recognized as a serious comorbidity in patients with HF and is associated with increased morbidity and mortality. In the present study intravenous FCM administration successfully repleted iron stores and improved symptoms and well-being in patients. Although FCM may have an effect on peak VO_2_, this cannot be conclusively determined from the present study because of the dependence of the finding on the imputation method.

The present findings extend the earlier reported favorable effect of FCM on 6-minute walking distance, symptoms, and other clinical parameters in patients with HF and iron deficiency.^9,10^ In the first FAIR-HF trial (Ferinject Assessment in Patients With Iron Deficiency and Chronic Heart Failure),^9^ 459 patients with HF with iron deficiency were enrolled, and intravenous FCM was shown to improve symptoms, quality of life parameters, and 6-minute walking distance test after 24 weeks in patients with and without anemia. In the second study in 304 patients with HF, the CONFIRM-HF (Ferric Carboxymaltose Evaluation on Performance in Patients With Iron deficiency in Combination With Chronic Heart Failure),^10^ treatment with FCM for 1 year resulted in a beneficial effect on symptoms, functional capacity by 6-minute walking test distance, and quality-of-life parameters. In addition, it also showed a reduction in the risk of first hospitalization for worsening HF compared with placebo (32 versus 10 events, *P*=0.009). In these 2 studies, FCM was well tolerated, and the data have led to a class IIa recommendation for intravenous FCM (ie, it should be considered in “symptomatic patients with HF and reduced LVEF, in order to alleviate HF symptoms, and improve exercise capacity and quality of life” in the 2016 Guidelines for HF of the European Society of Cardiology).^1^ Remarkably, iron deficiency is mentioned in neither the 2013 American College of Cardiology/American Heart Association Guidelines for the Management of Heart Failure^17^ nor in the 2016 Focused Update on New Pharmacological Therapy for Heart Failure.^18^

The mechanism of action of iron supplementation and exercise capacity in HF is still unclear. Besides its effect on Hb, iron has an important role in cellular oxygen storage and metabolism, especially in cells with a high energy demand such as cardiomyocytes and skeletal myocytes. In this process, mitochondria are the most important sites of iron utilization and energy production. Animal experiments show that iron deficiency results in impaired exercise capacity, and that substitution of iron leads to normalization of their exercise capacity independent of Hb. Such data, however, are not available in humans, but it is conceivable that iron supplementation affects either peripheral muscle or cardiac efficiency, but clearly more work is needed in this field.

Administration of oral iron is usually the first route to replete iron in patients with iron deficiency (with or without anemia), which is common worldwide.^19^ However, oral iron supplementation may not be useful in all patients with iron deficiency because it is often not absorbed well.^2,20^ In patients with chronic kidney disease, oral iron administration was unable to maintain adequate serum ferritin levels, in contrast to intravenous iron supplementation, and the latter was also superior with regard to the hematopoietic response.^20^ However, until recently, such data in patients with HF were scarce.^21^ Recently, the data of the IRONOUT HF study (Oral Iron Repletion Effect on Oxygen Uptake in Heart Failure), (NCT02188784)^22^ were published.^23^ In that study, 225 patients with symptomatic HF and iron deficiency were randomized to oral iron polysaccharide 150 mg bid or placebo, and the primary end point was change in peak VO_2_ after 16 weeks. Oral iron did not affect peak VO_2_ (or 6-minute walking distance) but also did not significantly increase serum ferritin and was apparently not able to adequately replete iron stores. The authors concluded that “these results do not support use of oral iron supplementation in patients with HF and reduced LVEF.”^23^

Measurement of peak VO_2_ during CPET is generally recognized as the gold standard of exercise capacity and a predictor of outcome in HF.^11,12,15,24^ It is more expensive and time-consuming than a simple exercise test, but it is superior in the assessment of severity of disease and outcome. Nevertheless, measurement of peak VO_2_ during CPET requires experienced staff. In the present study, all sites were well acquainted with CPET, and staff members were trained at the central core laboratory. Also, all patients had performed ≥2 (baseline) tests before randomization (which had to differ <10%), and we believe this has resulted in high-quality data. Having said this, we must point out that this was a controlled but open-label study, which may have caused bias. Indeed, despite that the CPET results were evaluated blindly in a core laboratory, knowledge of treatment assignment may have influenced the study. In the present study, we observed a difference between groups of 1.04 mL/kg/min (with LOCF analysis) in favor of FCM. Although this difference (7.5%) was smaller than expected, it may still be clinically relevant, and a recent study showed that every 6% change in peak VO_2_ was associated with a 5% difference in the primary end point of all-cause mortality and hospitalization.^24^

The handling of the deaths in the analysis of the present study deserves attention. Imputation of missing data in a study such as the present is notoriously difficult, and there is no perfect way to do this. The effect of FCM on peak VO_2_ would be less and not statistically significant if we had not imputed. However, death in HF trial is a real and objective finding, and by the steering committee agreed (and laid out in the statistical analysis plan) at the start of the study to do it this way. Also, ignoring the deaths by not imputing would have caused a selection and may lead to a biased estimation of the treatment effect. In contrast, hospitalizations for HF are much more difficult to assess and less objective, and large differences occur among countries in terms of criteria for hospital admission and organization. In general, the present study was clearly not powered to examine clinical events, and therefore the observed effects on deaths and hospitalizations must be interpreted with caution. Nevertheless, the total number of hospitalizations was higher in the FCM group (37 versus 21), which was mainly because of more hospitalizations for other cardiovascular reasons. An important part of these were because of elective procedures, which had been planned before enrollment (and which of course could have taken place before the study). The number of hospitalizations for worsening HF was similar in the 2 groups, but this occurred in slightly more patients on FCM. However, it is important to note that the total number of patients with clinically relevant events was in fact similar (11 versus 10 patients), with 4 deaths in the standard of care arm and no deaths in patients on FCM. In a recent meta-analysis,^25^ the effect of intravenous iron administration in patients with HF and iron deficiency on outcome was examined (from 5 controlled studies^9,10,14,16,26^) on 851 patients, of whom 509 received intravenous iron.^9,10,14,16,26^ In this meta-analysis, use of intravenous iron (versus controls) was associated with a reduction of the combined end points of all-cause mortality and cardiovascular hospitalizations (odds ratio, 0.44; *P*<0.0001), and this finding was largely driven by a reduction in HF hospitalizations (odds ratio for mortality, 0.83; *P*=0.56).^25^ The effect of FCM on outcome in HF will be investigated in 2 large trials: the AFFIRM-AHF (Study to Compare Ferric Carboxymaltose With Placebo in Patients With Acute Heart Failure and Iron Deficiency; NCT02937454; 1100 patients after a hospitalization for acute HF) and FAIR-HF 2^19^ (1200 patients with chronic HF).

In conclusion, in patients with chronic HF and iron deficiency, intravenous iron supplementation with FCM has a beneficial effect on peak VO_2_ compared with standard of care treatment, irrespective of the presence of baseline anemia. The effect of FCM on clinical outcome in this population merits further study.

## Acknowledgments

The authors dedicate this work to their wonderful and inspiring colleagues and fellow steering committee members, Vivian Conraads (Antwerp, Belgium) and Henry Krum (Melbourne, Australia), who died during the study. This study was presented in part at the Annual Scientific Sessions of the American Heart Association in November 2016 in New Orleans, LA.

## Sources of Funding

The study was sponsored by Vifor Pharma, Switzerland. Dr van Veldhuisen is an Established Investigator of The Netherlands Heart Foundation (grant D97.017).

## Disclosures

Drs van Veldhuisen, Ponikowski, van der Meer, Metra, Böhm, Doletsky, Voors, Macdougall, Anker, Filippatos, and Cohen-Solal all received research funding, consultancy fees, or honoraria from Vifor. Drs Zakin and Roubert are employees of Vifor.

## Supplementary Material

## References

[1] Ponikowski P, Voors AA, Anker SD, Bueno H, Cleland JG, Coats AJ, Falk V, González-Juanatey JR, Harjola VP, Jankowska EA, Jessup M, Linde C, Nihoyannopoulos P, Parissis JT, Pieske B, Riley JP, Rosano GM, Ruilope LM, Ruschitzka F, Rutten FH, van der Meer P, Authors/Task Force Members (2016). 2016 ESC Guidelines for the diagnosis and treatment of acute and chronic heart failure: the Task Force for the diagnosis and treatment of acute and chronic heart failure of the European Society of Cardiology (ESC) developed with the special contribution of the Heart Failure Association (HFA) of the ESC.. Eur Heart J.

[2] Van Veldhuisen DJ, Anker SD, Ponikowski P, Macdougall IC (2011). Anaemia and iron deficiency in heart failure: mechanisms and therapeutic applications.. Nat Rev Cardiol.

[3] Klip IT, Comin-Colet J, Voors AA, Ponikowski P, Enjuanes C, Banasiak W, Lok DJ, Rosentryt P, Torrens A, Polonski L, van Veldhuisen DJ, van der Meer P, Jankowska EA (2013). Iron deficiency in chronic heart failure: an international pooled analysis.. Am Heart J.

[4] Okonko DO, Mandal AK, Missouris CG, Poole-Wilson PA (2011). Disordered iron homeostasis in chronic heart failure: prevalence, predictors, and relation to anemia, exercise capacity, and survival.. J Am Coll Cardiol.

[5] Cleland JG, Zhang J, Pellicori P, Dicken B, Dierckx R, Shoaib A, Wong K, Rigby A, Goode K, Clark AL (2016). Prevalence and outcomes of anemia and hematinic deficiencies in patients with chronic heart failure.. JAMA Cardiol.

[6] Brownlie T, Utermohlen V, Hinton PS, Haas JD (2004). Tissue iron deficiency without anemia impairs adaptation in endurance capacity after aerobic training in previously untrained women.. Am J Clin Nutr.

[7] Jankowska EA, von Haehling S, Anker SD, Macdougall IC, Ponikowski P (2013). Iron deficiency and heart failure: diagnostic dilemmas and therapeutic perspectives.. Eur Heart J.

[8] Cohen-Solal A, Leclercq C, Deray G, Lasocki S, Zambrowski JJ, Mebazaa A, de Groote P, Damy T, Galinier M (2014). Iron deficiency: an emerging therapeutic target in heart failure.. Heart.

[9] Anker SD, Comin Colet J, Filippatos G, Willenheimer R, Dickstein K, Drexler H, Lüscher TF, Bart B, Banasiak W, Niegowska J, Kirwan BA, Mori C, von Eisenhart Rothe B, Pocock SJ, Poole-Wilson PA, Ponikowski P, FAIR-HF Trial Investigators (2009). Ferric carboxymaltose in patients with heart failure and iron deficiency.. N Engl J Med.

[10] Ponikowski P, van Veldhuisen DJ, Comin-Colet J, Ertl G, Komajda M, Mareev V, McDonagh T, Parkhomenko A, Tavazzi L, Levesque V, Mori C, Roubert B, Filippatos G, Ruschitzka F, Anker SD, CONFIRM-HF Investigators (2015). Beneficial effects of long-term intravenous iron therapy with ferric carboxymaltose in patients with symptomatic heart failure and iron deficiency.. Eur Heart J.

[11] Malhotra R, Bakken K, D’Elia E, Lewis GD (2016). Cardiopulmonary exercise testing in heart failure.. JACC Heart Fail.

[12] Guazzi M, Adams V, Conraads V, Halle M, Mezzani A, Vanhees L, Arena R, Fletcher GF, Forman DE, Kitzman DW, Lavie CJ, Myers J, EACPR/AHA joint scientific statement (2012). Clinical recommendations for cardiopulmonary exercise testing data assessment in specific patient populations.. Eur Heart J.

[13] Tabet JY, Beauvais F, Thabut G, Tartière JM, Logeart D, Cohen-Solal A (2003). A critical appraisal of the prognostic value of the VE/VCO2 slope in chronic heart failure.. Eur J Cardiovasc Prev Rehabil.

[14] Keteyian SJ, Patel M, Kraus WE, Brawner CA, McConnell TR, Pina IL, Seifer ES, Fleg JL, Blackburn G, Fonarow GC, Chase PJ, Piner L, Vest M, O’Connor CM, Ehrmann JK, Walsh MN, Ewald G, Bensimhon D, Russell SD, on behalf of the HF-ACTION Investigators (2016). Variables measured during cardiopulmonary exercise testing as predictors of prognosis of mortality in chronic systolic heart failure.. J Am Coll Cardiol.

[15] van Veldhuisen DJ, Dickstein K, Cohen-Solal A, Lok DJ, Wasserman SM, Baker N, Rosser D, Cleland JG, Ponikowski P (2007). Randomized, double-blind, placebo-controlled study to evaluate the effect of two dosing regimens of darbepoetin alfa in patients with heart failure and anaemia.. Eur Heart J.

[16] Okonko DO, Grzeslo A, Witkowski T, Mandal AK, Slater RM, Roughton M, Foldes G, Thum T, Majda J, Banasiak W, Missouris CG, Poole-Wilson PA, Anker SD, Ponikowski P (2008). Effect of intravenous iron sucrose on exercise tolerance in anemic and nonanemic patients with symptomatic chronic heart failure and iron deficiency FERRIC-HF: a randomized, controlled, observer-blinded trial.. J Am Coll Cardiol.

[17] Yancy CW, Jessup M, Bozkurt B, Butler J, Casey DE, Drazner MH, Fonarow GC, Geraci SA, Horwich T, Januzzi JL, Johnson MR, Kasper EK, Levy WC, Masoudi FA, McBride PE, McMurray JJV, Mitchell JE, Peterson PN, Riegel B, Sam F, Stevenson LW, Tang WHW, Tsai EJ, Wilkoff BL (2013). 2013 ACCF/AHA Guideline for the management of heart failure: a report of the American College of Cardiology Foundation/American Heart Association Task Force on Practice Guidelines.. Circulation.

[18] Yancy CW, Jessup M, Bozkurt B, Butler J, Casey DE, Colvin MM, Drazner MH, Filippatos G, Fonarow GC, Givertz MM, Hollenberg SM, Lindenfeld JA, Masoudi FA, McBride PE, Peterson PN, Stevenson LW, Westlake C (2016). 2016 ACC/AHA/HFSA focused update on new pharmacological therapy for heart failure: an update of the 2013 ACCF/AHA Guideline for the management of heart failure: a report of the American College of Cardiology/American Heart Association Task force on Clinical Practice Guidelines and the Heart Failure Society of America.. Circulation.

[19] von Haehling S, Jankowska EA, van Veldhuisen DJ, Ponikowski P, Anker SD (2015). Iron deficiency and cardiovascular disease.. Nat Rev Cardiol.

[20] Macdougall IC, Tucker B, Thompson J, Tomson CR, Baker LR, Raine AE (1996). A randomized controlled study of iron supplementation in patients treated with erythropoietin.. Kidney Int.

[21] Beck-da-Silva L, Piardi D, Soder S, Rohde LE, Pereira-Barretto AC, De Albuquerque D, Bocchi E, Vilas-Boas F, Moura LZ, Montera MW, Rassi S, Clausell N (2013). IRON-HF study: a randomized trial to assess the effects of iron in heart failure patients with anemia.. Int J Cardiol.

[22] Lewis GD, Semigran MJ, Givertz MM, Malhotra R, Anstrom KJ, Hernandez AF, Shah MR, Braunwald E, Oral iron therapy for heart failure with reduced ejection fraction (2016). Design and rationale for Oral Iron Repletion Effects on Oxygen Uptake in Heart Failure (IRONOUT HF).. Circ Heart Fail.

[23] Lewis GD, Malhotra R, Hernandez AF, McNulty SE, Smith A, Felker GM, Tang WHW, LaRue SJ, Redfield MM, Semigran MJ, Givertz MM, Van Buren P, Whellan D, Anstrom KJ, Shah MR, Desvigne-Nickens P, Butler J, Braunwald E, NHLBI Heart Failure Clinical Research Network (2017). Effect of Oral Iron Repletion on Exercise Capacity in Patients With Heart Failure With Reduced Ejection Fraction and Iron Deficiency: The IRONOUT HF randomized clinical trial.. JAMA.

[24] Swank AM, Horton J, Fleg JL, Fonarow GC, Keteyian S, Goldberg L, Wolfel G, Handberg EM, Bensimhon D, Illiou MC, Vest M, Ewald G, Blackburn G, Leifer E, Cooper L, Kraus WE, HF-ACTION Investigators (2012). Modest increase in peak VO_2_ is related to better clinical outcomes in chronic heart failure patients: results from heart failure and a controlled trial to investigate outcomes of exercise training.. Circ Heart Fail.

[25] Jankowska EA, Tkaczyszyn M, Suchocki T, Drozd M, von Haehling S, Doehner W, Banasiak W, Filippatos G, Anker SD, Ponikowski P (2016). Effects of intravenous iron therapy in iron-deficient patients with systolic heart failure: a meta-analysis of randomized controlled trials.. Eur J Heart Fail.

[26] Toblli JE, Lombrana A, Duarte P, Di Genarro F (2007). Intravenous iron reduces NT-pro-brain natriuretic peptide in anemic patienst with chronic heart failure and iron deficiency.. J Am Coll Cardiol.

