# Supplementary material for: Effect of Ferric Carboxymaltose on Exercise Capacity in Patients With Chronic Heart Failure and Iron Deficiency
Source: Circulation. 2017 Oct 9;136(15):1374–83. doi: 10.1161/CIRCULATIONAHA.117.027497 (PMC5642327; doi:10.1161/CIRCULATIONAHA.117.027497)

SUPPLEMENTAL MATERIAL

Appendix

Participants of the EFFECT-HF study:

Steering Committee: Dirk J. van Veldhuisen (chair); Piotr Ponikowski (co-chair), Alain Cohen-Solal, Michael Böhm, Marco Metra, Peter van der Meer, Artem Doletsky, Adriaan A. Voors, Iain Macdougall, and Viviane Conraads and Henry Krum.

Data Safety and Monitoring Board: Henry Dargie (chair), Rudolf Wuthrich, and Peter Shabe (Statistician). Endpoint Adjudication Committee: Stefan D. Anker (Chair), Gerasimos Filippatos, Frank Ruschitzka.

Supplementary Figure 1: **Association between the change in Peak VO_2 and the change in Hemoglobin at week 24 (LOCF)**.

There was no association between the effect of FCM on Peak VO_2 and the effect on Hemoglobin ($r=-0.0758$, $p=0.51$).

Ferinject Protocol FER-CARS-04 **Page 1 of 1**

AHA Figure 5.1 Change in Peak VO2 [ml/kg/min] by Change in Haemoglobin [g/dL] at Week 24 (LOCF)

Pearson correlation coefficient - FCM: r=-0.0758, p=0.5039 / Standard of Care: r=-0.1147, p=0.3142

Analysis Set: Full Analysis Set

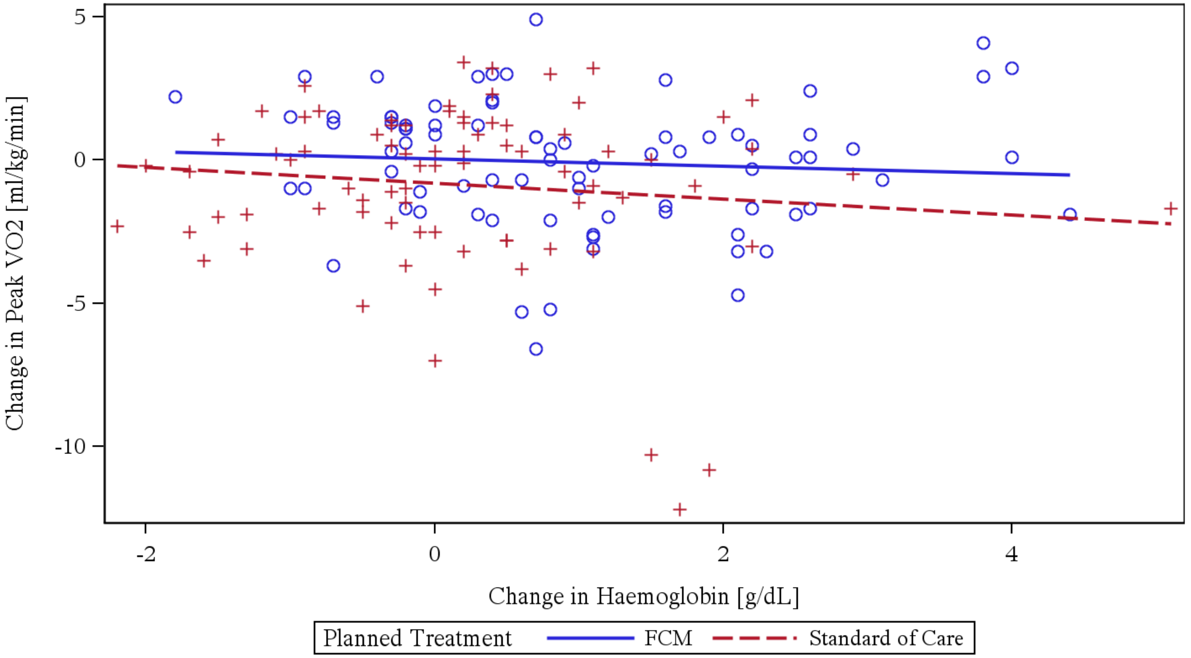

Regression lines for each treatment group are displayed.
Source datasets: ADSL, ADLB, ADEFF; Program: AHA PHF5 x.sas; Output: aha phf 5 1.rtf; Generated on: 25OCT16 : 15:30

Supplementary Figure 2: **Effect of FCM on NYHA functional class**

Values are changes compared to baseline and relative to the change in the control (standard of care) group, whereby an Odds ratio (OR) > 1.0 means effect in the FCM group is better than the standard of care group.

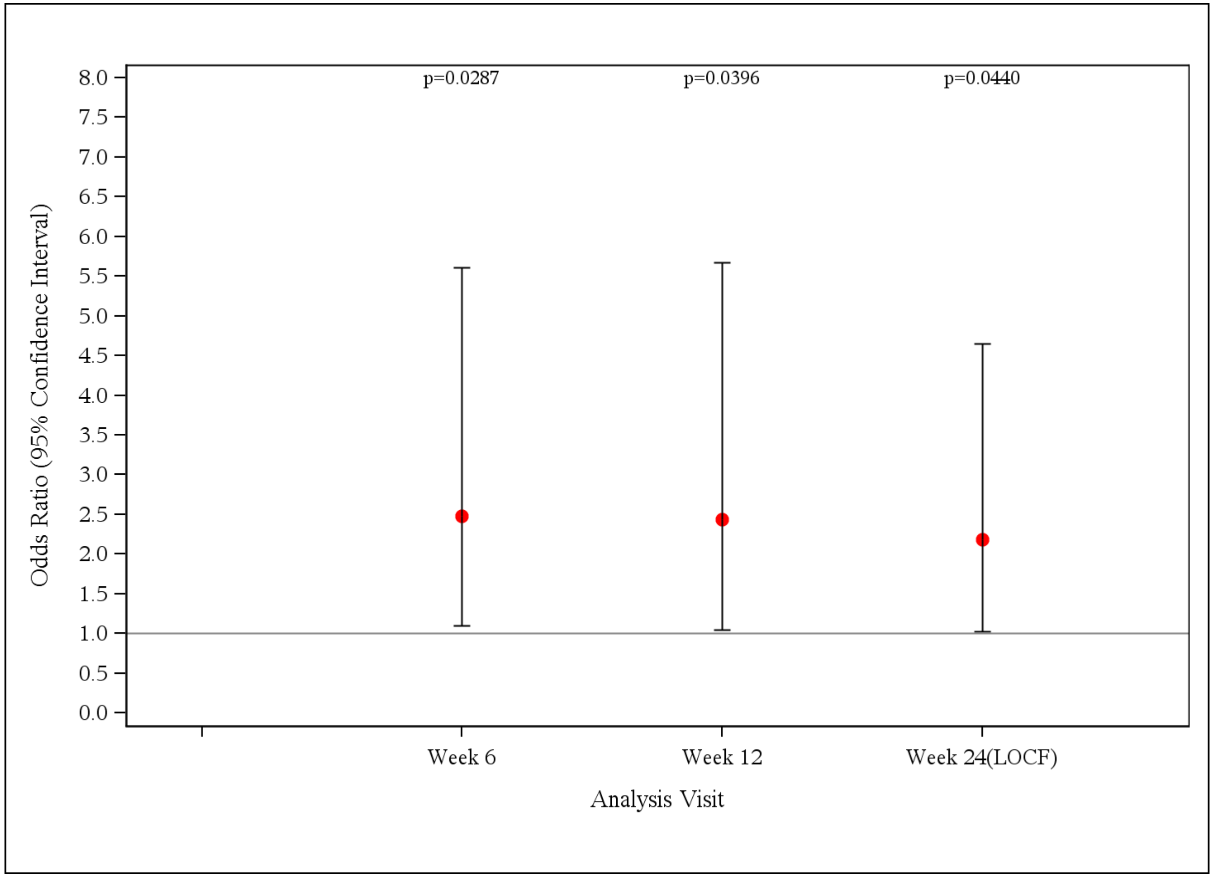

Supplementary Figure 3: **Effect of FCM on PGA score.**

Values are changes compared to baseline and relative to the change in the control (standard of care) group, whereby an Odds ratio (OR) > 1.0 means effect in the FCM group is better than the standard of care group.

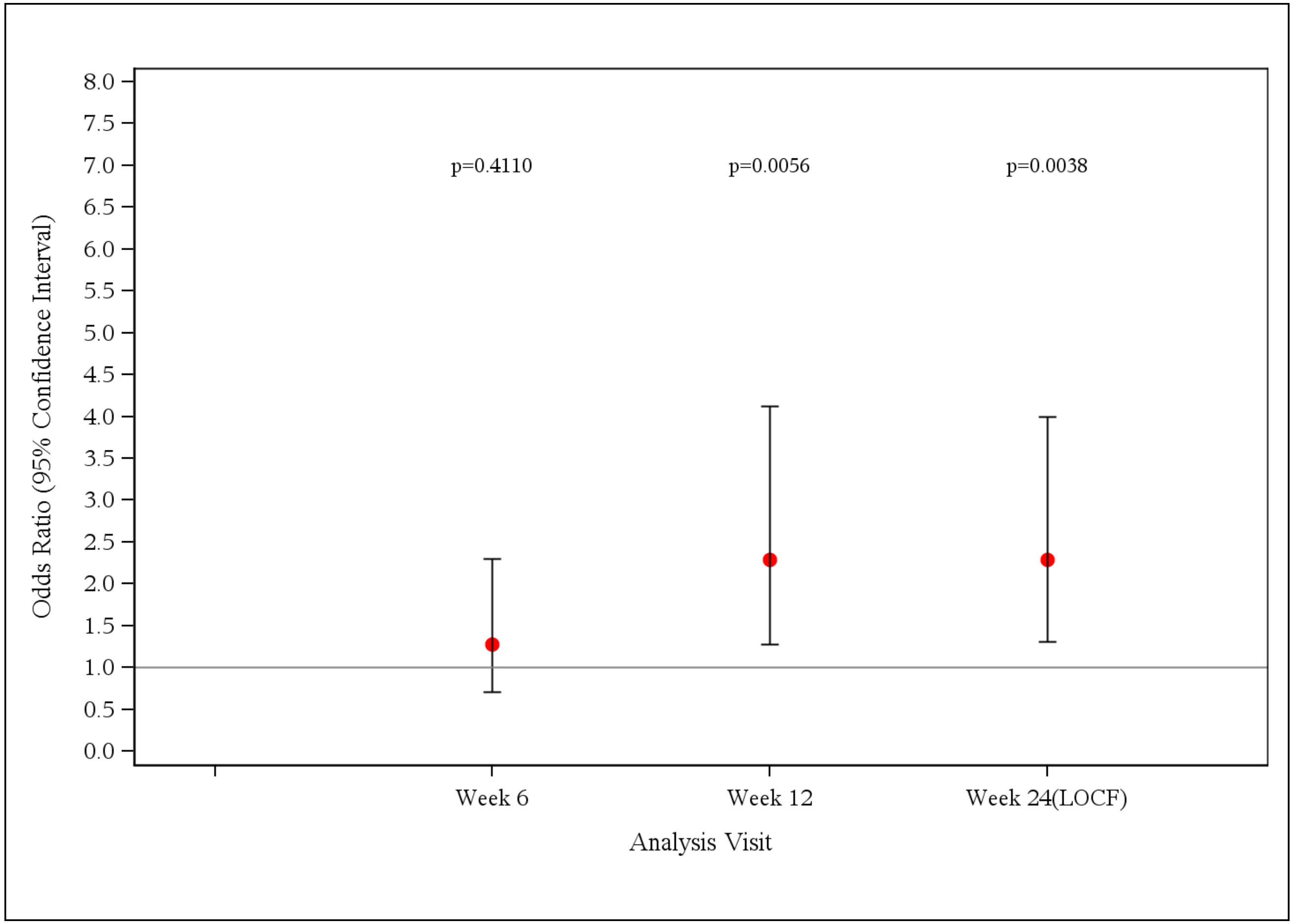

Supplement: Supplementary file 1 [file cir-136-1374-s001.pdf]
